# Building a dementia‐capable workforce: Implementation and evaluation of a state‐mandated dementia training program for memory care staff

**DOI:** 10.1002/alz.71813

**Published:** 2026-08-31

**Authors:** Sara Crance, Gillian Hamilton, Calli Carlson, Kellie Garrett, Tory Roberg

**Affiliations:** ^1^ Hospice of the Valley's Dementia Care and Education Campus Phoenix Arizona USA; ^2^ Arizona State University Edson College of Nursing and Health Innovation Phoenix Arizona USA; ^3^ Southwest and Mountain Territory Alzheimer's Association Phoenix Arizona USA

**Keywords:** dementia care, health policy, memory care, state legislation, workforce training

## Abstract

As dementia prevalence rises, developing a dementia‐capable workforce is a national priority. Training requirements vary widely across states, and many memory care staff receive limited dementia education. Although evidence supports dementia training, little is known about translating policy into practice. This paper describes the implementation and evaluation of a statewide mandated dementia training initiative in Arizona. After passage of AZ HB2764 (2024), developed and supported by the Alzheimer's Association Arizona Chapter, an interdisciplinary team at Hospice of the Valley developed a state‐approved program including 6 hours of online modules and a 2‐hour in‐person training. From August to December 2025, 3409 staff across diverse roles completed the program. Knowledge scores improved (79.74%–95.97%), with high satisfaction (4.89/5); 98.53% reported role applicability, and 94.6% reported improved care ability at 3 months. Findings support mandated, statewide dementia training as a feasible policy strategy to strengthen workforce capacity and improve dementia‐capable care.

## 1 BACKGROUND

As the prevalence of Alzheimer's disease and related dementias (ADRD) continues to increase in the United States, the need for a dementia‐capable workforce has become a national priority.^1^, ^2^, ^3^, ^4^ Individuals living with dementia experience progressive cognitive and functional changes that impact daily life.^5^, ^6^ Assisted living facilities that provide memory care are specifically designed to serve individuals living with dementia by providing safe, structured supervision, care, and environmental support. However, the quality of care delivered in these environments depends largely on the knowledge, skills, and experience of caregivers and staff. Direct care staff provide the majority of hands‐on care and assistance, while other staff interact regularly with residents. Their ability to recognize cognitive impairment; interpret expressions and responses; and implement supportive, non‐pharmacologic interventions is crucial to resident safety and quality of life.^7^

Regulatory requirements for dementia‐specific training vary widely across states, with some mandating structured education in training programs and in facilities and continuing competency requirements, while other states require only minimal facility orientation.^3^, ^8^, ^9^, ^10^ Analyses of the national workforce further demonstrate that staff with the most direct resident contact often receive the least formal dementia education.^4^ As a result, staff are expected to manage complex situations without adequate preparation, and many report feeling underprepared to care for residents with dementia and express a desire for additional training.^11^, ^12^

Insufficient staff training leads to decreased staff confidence, burnout, and measurable clinical outcomes, such as misinterpreting resident responses and managing symptoms pharmacologically rather than through supportive interventions. Two studies of nursing homes with state‐mandated dementia training above federal minimal standards demonstrate lower rates of inappropriate psychotropic medication use, suggesting caregiver knowledge may directly influence or impact behavioral management practices.^13^, ^14^ Structured dementia education training programs have also been associated with improved communication, fewer behavioral crises, and increased staff confidence in care delivery.^10^, ^15^ This evidence suggests dementia training functions not only as professional education and training, but also as a quality and safety intervention within long‐term care settings.^16^ Prior research identifies regulatory variability and workforce training gaps, while less attention has been given to how policy initiatives can be translated into practical education training programs and implemented into real‐world care environments.^9^

The purpose of this paper is to describe a statewide dementia workforce initiative in Arizona, including the policy process leading to mandated dementia training for all staff at assisted living facilities that provide memory care, the development and implementation of a state‐approved training program, and the outcomes observed during and after the training. This initiative may serve as a practical model for other states seeking to improve their dementia‐capable workforce through training and regulatory policy.

### 1.1 Policy development and legislative process

In 2023, *The Arizona Republic* newspaper published a year‐long investigative series examining problems in senior living facilities across Arizona. Several widely publicized stories documented incidents in which residents injured or killed one another when caregivers lacked the training necessary to manage symptoms associated with dementia. The investigation highlighted an urgent need for reform in memory care services. At that time, > 1800 facilities in Arizona licensed to provide “directed care services” (care for individuals unable to direct their own care due to dementia) marketed themselves as “memory care” facilities. However, these facilities operated under minimal and inconsistent regulatory standards, as there were no existing memory care training requirements, and were often limited to the requirement of a secured or locked door.

In response to hundreds of emails and phone calls from concerned families, the Alzheimer's Association Arizona Chapter undertook the development of proposed standards for memory care in an effort to raise the quality of care across the state. The organization conducted listening sessions with families and providers, reviewed current research, examined best practices from other states, and collaborated with stakeholders to identify areas of consensus for potential legislation. These efforts were further informed by the Alzheimer's Association Dementia Care Practice Recommendations, which outline evidence‐based, person‐centered care standards to guide quality dementia care practice across settings.^17^ Over the following year, the Association worked with members of both the Arizona Senate and House of Representatives to develop legislation. The resulting bill, Arizona House Bill 2764 (2024), underwent multiple revisions before being signed into law by Governor Katie Hobbs in April 2024.

After the bill's passage, the Arizona Department of Health Services convened a series of stakeholder meetings to gather additional input from industry representatives and patient advocates regarding implementation of the regulations. The final regulatory review occurred during a meeting of the Governor's Regulatory Review Council on June 3, 2025. The meeting was contentious, with significant opposition from industry representatives who argued that the proposed training requirements would be burdensome, costly, and duplicative. However, with the encouragement of the Alzheimer's Association, families affected by inadequate care were also present and provided powerful testimony describing abuse and neglect experienced by their loved ones in facilities. One speaker recounted the death of her mother, who was fatally beaten by another resident in a memory care setting. As one rural assisted living manager noted during the discussion, many caregivers lack a fundamental understanding of dementia and may misinterpret symptoms as intentional misbehavior, sometimes responding with verbal or physical punishment.

After nearly 2 years of development and debate, the regulations were ultimately approved. A central component of the new rules is the requirement for a comprehensive memory care training program applicable not only to designated caregivers but to all staff members who interact with residents.

### 1.2 Development of the dementia training program

**FIGURE 1:**
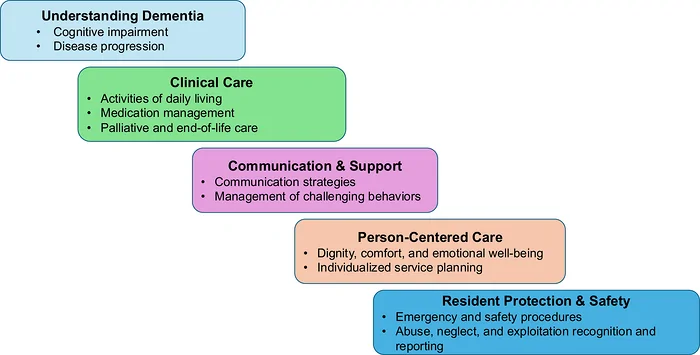
Arizona state‐mandated memory care training curriculum.

To deliver the state‐mandated dementia training, organizations must apply to and receive approval from the Arizona Department of Health Services (AZDHS). Approval requires demonstrating that the program meets state requirements for content, instruction, and certificate issuance, as outlined in R9‐10‐122.^18^ After the enactment of the training requirement and meetings with the AZDHS, Hospice of the Valley's team at the Dementia Care and Education Campus began developing a memory care training program to be offered at no cost in compliance with the requirements. AZDHS requires 8 hours of training, including an in‐person component that demonstrates the individual's skills and knowledge. In accordance to R9‐10‐122,^18^ all programs are required to address the following topics: understanding cognitive impairments and their impact on residents, including the progression of neurodegenerative disease; communication techniques for individuals with cognitive impairment; management of challenging behaviors such as aggression, wandering, and agitation; strategies to promote dignity, comfort, and emotional well‐being; implementation of individualized service planning for residents; emergency and safety protocols specific to memory care; recognition, prevention, and reporting of abuse, neglect, or exploitation; activities of daily living specific to memory care residents; palliative care and end‐of‐life training; and medication management and administration (see Figure 1). Hospice of the Valley is one of 125 training programs currently approved by the AZDHS to provide the mandated dementia training to assisted living staff providing memory care services at > 1800 facilities throughout the state.

Hospice of the Valley's curriculum was developed by a multidisciplinary team, including clinicians (licensed/registered occupational therapists, master of social work, doctor, nurse practitioner) with experience in dementia care and education. Both lead trainers obtained their Certified Dementia Practitioner certifications from the National Council of Certified Dementia Practitioners to meet the trainer requirements. The required topics were divided, and a review of evidence‐based research was conducted for each area. The curriculum development process was flexible, allowing content length to vary based on topic, with modules ranging from 30 to 60 minutes. Once content development was complete, the team prepared a final manuscript and formatted the materials into a learner‐friendly training packet. The curriculum was then reviewed and edited by other members of the multidisciplinary team. After content completion, scenarios and education were filmed for each training module, totaling ≈ 6 hours of recorded content. A learning management system (LMS) assessment was developed with questions to track learner progress throughout each module. All training materials were submitted to AZDHS for approval on July 1, 2025, and approval for the program was received on July 7, 2025.

For the in‐person component, to be delivered after the modules were completed with satisfactory passing scores, the multidisciplinary Hospice of the Valley team identified curriculum elements best suited for live instruction. To support trainer feasibility and minimize time away from facility staff's work areas, the in‐person training was designed as a 2‐hour session. To promote consistency across training sessions, all trainers used a standardized curriculum and instructional materials. The 2‐hour in‐person training included an interactive experience called Dementia Moments to simulate the emotional perspective of individuals living with dementia using headphones, goggles, gloves, and instructions to complete five task‐based activities; hands‐on mobility demonstration including ambulation and transfer practice; role‐playing approach techniques to address visual changes; communication strategies for responding to distress; and evacuation and elopement procedures specific to memory care residents. Participant engagement was assessed through instructor observation during communication, mobility, and disaster‐response simulations. Instructors circulated throughout the room to monitor participation and interactions while providing feedback. Participants were required to arrive on time and complete the end‐of‐session evaluation to receive their certificate of completion.

## 2 RESULTS

### 2.1 Training participation

A total of *n* = 3409 participants completed both the online and in‐person training program between August and December 2025, with data presented below. (The training continued subsequently, with ≈ 300 participants trained monthly.) Participants represented facilities throughout Arizona and included roles such as caregiver, certified nursing assistant, nurse, administrator, assisted living manager, culinary, maintenance, activities, housekeeping, medical providers, receptionist, security, and chaplain. An additional 4 hours of training were required for assisted living managers: 3 hours were delivered by module, and 1 hour was a live Zoom educational session. The manager training topics included development and implementation of individualized service plans, staffing levels and resource allocation, identification and implementation of control measures for infectious disease, and developing emergency protocols.

### 2.2 Knowledge improvement

Across the 10 online modules, mean pre‐training knowledge scores were 79.74%, increasing to 95.97% after completing the training module. Pre‐ and post‐tests consisted of 40 questions across the 10 modules, written at an 8th‐grade reading level. Post‐tests were required immediately after the module content, and completion was required to progress to the next module. All modules demonstrated improvement in post‐test scores (see Table 1). Overall knowledge scores improved by 16.23 percentage points, indicating improved knowledge of dementia care concepts after completion of the required education.

**TABLE 1:** Pre‐test/post‐test averages by module.

Module	Pre‐test average	Post‐test average
Module 1: Understanding cognitive impairments	73.01%	95.18%
Module 2: Communication techniques	75.97%	98.01%
Module 3: Managing challenging behaviors	73.04%	95.34%
Module 4: Techniques for promoting dignity, comfort, and emotional well‐being of residents	73.95%	93.66%
Module 5: Implementation of individualized service planning	77.71%	93.28%
Module 6: Emergency and safety protocols specific to memory care	87.19%	97.14%
Module 7: Recognizing, preventing, and reporting abuse, neglect, or exploitation	90.45%	97.44%
Module 8: Activities of daily living specific to residents receiving memory care services	77.25%	94.99%
Module 9: Palliative care and end‐of‐life training	80.63%	96.46%
Module 10: Medication management and administration	88.19%	98.16%

### 2.3 Immediate impact of the training

After completion of the in‐person training session, participants reported increased preparedness to care for and interact with residents with dementia. The majority of participants (98.53%) agreed or strongly agreed that the dementia training content was applicable to their work. Additionally, among the 3409 attendees who completed the training between August and December 2025, the overall experience was highly favorable, with a mean score of 4.89 on a 5‐point scale (1 = poor; 5 = excellent).

Responses to open‐ended questions further supported these findings. In open‐ended questions regarding the most important concept learned from the modules, participants shared approach and communication techniques, recognition and ability to address mobility challenges, the importance of empathy and patience, and techniques for providing person‐centered care. Additional feedback included how beneficial the training was, how applicable it was to the individual's role in the facility, the opportunity to learn new approaches and skills even with years of dementia care experience, and the desire for continued dementia education and training.

“The training was amazing, I never thought that I would deal with a resident since I am a cook, but knowing these skills is helpful anyways.”

“Extremely amazing training, gave me a whole different perspective of what they [residents] deal with and how I become a huge impact on their behavior.”

“I'm just a security officer, but sometimes residents come up to me with a handful of lightbulbs, and I have no idea what to say: now I do! Thank you!”

“I really loved it. It opened my eyes to a lot of new things that I am going to apply to my work and just day to day. Thank you so much for the time and the knowledge!”

“I thought the training was excellent. And I have about 30 caregivers that work for me and almost all of them had very positive feedback and they were excited about the training and engaged. So, thank you!”

### 2.4 Practice change

A 3‐month follow‐up survey was distributed via e‐mail to training participants to assess the impact of the dementia training on dementia care practices. Among the respondents (*n* = 167), 94.6% strongly agreed or agreed that the training had been helpful in caring for individuals living with dementia, with the majority (83.2%) strongly agreeing. Open‐ended responses further illustrated the perceived impact of the training on the participants’ daily work. When asked how they had applied the training in practice, respondents reported feeling better equipped and more prepared to work with, communicate with, and care for individuals living with dementia. Participants also described having an increased sense of empathy and compassion toward residents and noted that the training helped them better recognize and respond to the needs of individuals living with dementia.

“I have been a caregiver for 15 years and I've always tried to meet my residents as if they were my own family… This training has made me put myself in their shoes and see from their frustrations and irritations and why they get upset which I never really did until the class.”

“When I'm working directly with my resident, I'm more confident now on how to redirect them so the behavior doesn't escalate.”

“I have been able to use the techniques to approach with better attention and understanding.”

“Instead of arguing with a resident who was distressed because they thought they needed to pick up their children from school, I used validation therapy to acknowledge their feelings of responsibility. I said ‘you have been a wonderful parent’ then I used redirection by asking them to help me fold laundry which brought them a sense of purpose and calmed their anxiety.”

## 3 DISCUSSION

To our knowledge, this is the first paper to describe and evaluate a mandatory state‐wide dementia‐specific training for all staff, not just direct care staff, who come in contact with residents of assisted living facilities that provide memory care. One study^15^ focused on 209 dyads of California Medicaid recipients, in which the in‐home paid caregivers (66% family members, 11% friends and neighbors, 23% paid professionals) participated in an entirely voluntary virtual training. Mixed results were found regarding its effects on their care. No study has evaluated wide‐scale training outside of nursing homes or home care.

This paper demonstrates how regulatory policy mandating dementia training and the implementation of a dementia training program can support improvements in dementia care knowledge and workforce preparedness for staff in assisted living facilities that provide memory care. Findings demonstrate that a regulatory dementia training requirement can be operationalized within real‐world settings to help improve knowledge and preparedness for working with individuals living with dementia. These results suggest that mandated workforce education may represent a feasible policy approach to strengthening workforce capability in memory care environments, with the potential to support improvements in dementia care.

Participants demonstrated measurable improvements in dementia‐related knowledge across all online training modules. These findings are consistent with prior research demonstrating that structured dementia education programs improve knowledge, communication skills, and competence in managing dementia‐related behaviors and responses.^10^, ^14^, ^15^, ^16^ Improvements were acknowledged across key competencies for dementia care, including communication and approach techniques, behavioral management, support with activities of daily living, and safety protocols through evacuation and elopement preparation drills.

The training was also reported to be highly applicable to participant roles within the facilities, including staff members whose primary responsibilities were not direct caregiving (e.g., maintenance, culinary, security, etc.). This finding highlights the importance of educating the broader memory care facility workforce, as residents living with dementia interact with diverse staff positions within the care environment and are not only limited to direct care staff. Previous research suggests that dementia care quality is shaped by everyday interactions in the care environment and not just clinical care.^7^ Training all memory care staff has the potential to support more consistent dementia‐capable environments.

Feedback and follow‐up survey responses further indicate that participants reported positive changes in practice after dementia training. Participants described increased confidence in their ability to communicate with residents, use validation and redirection techniques, and respond to behavioral responses in ways that decreased distress. Although a comparison group was not included, the training team reported that the in‐person session, which incorporated active simulation and participant role‐playing after module completion, played a critical role in reinforcing learning and promoting engagement in applying these care approaches.

The findings from this paper also highlight the broader policy implications of dementia workforce training initiatives. Prior research suggests that facilities located in states with enhanced mandated dementia training requirements in nursing homes demonstrate lower rates of inappropriate psychotropic medication use among residents with dementia.^13^, ^14^ While previous studies have documented variability in state dementia training requirements and workforce preparedness,^8^, ^9^ fewer have described how policy initiatives can be translated into practical education programs within real‐world care environments. This Arizona initiative demonstrates how collaboration among regulatory agencies, clinical experts, and educational organizations can operationalize regulatory requirements into scalable training programs capable of reaching a large, diverse dementia workforce.

## 4 LIMITATIONS AND IMPLICATIONS

Several limitations should be considered when examining the findings of this paper. Participation in the follow‐up survey was voluntary and may reflect response bias, as individuals with a positive training experience may have been more likely to respond. Additionally, outcomes were based primarily on knowledge assessments and self‐reported practice changes rather than observed clinical outcomes or behaviors. Future research should examine whether dementia workforce training initiatives are associated with measurable improvements in the lives of individuals living with dementia, including behavioral symptom management, safety, and quality of life. Additional research should examine the impact of mandatory dementia training on memory care staff, including confidence, job satisfaction, burnout, and turnover rates. Despite these limitations, this dementia training program reached > 4200 participants over 7 months across multiple roles within assisted living facilities that provide memory care throughout the state of Arizona. These findings suggest that regulatory policy, combined with accessible training programs, may provide an effective strategy for strengthening dementia workforce knowledge and capability and improving dementia care delivery within long‐term memory care environments.

## 5 CONCLUSION

As the prevalence of dementia continues to increase worldwide, strengthening the dementia care workforce is considered an essential component of supporting high‐quality dementia care in long‐term care environments.^1^, ^2^, ^3^, ^4^ This statewide dementia workforce initiative in Arizona demonstrates how regulatory policy, interdisciplinary collaboration, and structured education programs can be combined to support improvements in dementia care knowledge, workforce preparedness, and staff confidence in care delivery. The results suggest that mandated dementia training programs serve not only as ongoing professional education and training, but may also represent a quality improvement strategy with the potential to support more person‐centered care practices and safer care environments for residents living with dementia. By translating policy requirements into accessible training programs that include staff across multiple roles within memory care facilities, this initiative provides a practical model for other states seeking to enhance dementia workforce capability. Continued evaluation of workforce education initiatives will be important to determine their long‐term impact on care practices, staff outcomes, and the quality of life of individuals living with dementia (Supporting information).

## CONFLICT OF INTEREST STATEMENT

The authors declare no conflicts of interest. Author disclosures are available in the Supporting Information.

## Supporting information




**Supporting Information**: alz71813‐sup‐0001‐ICMJE.pdf
